# Isolation and characterization of a virus (CvV-BW1) that infects symbiotic algae of *Paramecium bursaria *in Lake Biwa, Japan

**DOI:** 10.1186/1743-422X-7-222

**Published:** 2010-09-13

**Authors:** Ryo Hoshina, Mayumi Shimizu, Yoichi Makino, Yoshihiro Haruyama, Shin-ichiro Ueda, Yutaka Kato, Masahiro Kasahara, Bun-ichiro Ono, Nobutaka Imamura

**Affiliations:** 1 Department of Biomedical Science, College of Life Sciences, Ritsumeikan University, Noji Higashi 1-1-1, Kusatsu, 525-8577 Japan; 2 Department of Bioscience and Biotechnology, Faculty of Science and Engineering, Ritsumeikan University, Noji Higashi 1-1-1, Kusatsu, 525-8577 Japan; 3 Department of Biotechnology, College of Life Sciences, Ritsumeikan University, Noji Higashi 1-1-1, Kusatsu, 525-8577 Japan; 4 Department of Pharmacy, College of Pharmaceutical Sciences, Ritsumeikan University, Noji Higashi 1-1-1, Kusatsu, 525-8577 Japan

## Abstract

**Background:**

We performed an environmental study of viruses infecting the symbiotic single-celled algae of *Paramecium bursaria *(*Paramecium bursaria Chlorella *virus, PBCV) in Lake Biwa, the largest lake in Japan. The viruses detected were all *Chlorella variabilis *virus (CvV = NC64A virus). One of them, designated CvV-BW1, was subjected to further characterization.

**Results:**

CvV-BW1 formed small plaques and had a linear DNA genome of 370 kb, as judged by pulsed-field gel electrophoresis. Restriction analysis indicated that CvV-BW1 DNA belongs to group H, one of the most resistant groups among CvV DNAs. Based on a phylogenetic tree constructed using the *dnapol *gene, CvV was classified into two clades, A and B. CvV-BW1 belonged to clade B, in contrast to all previously identified virus strains of group H that belonged to clade A.

**Conclusions:**

We conclude that CvV-BW1 composes a distinct species within *C. variabilis *virus.

## Background

**Figure 1:**
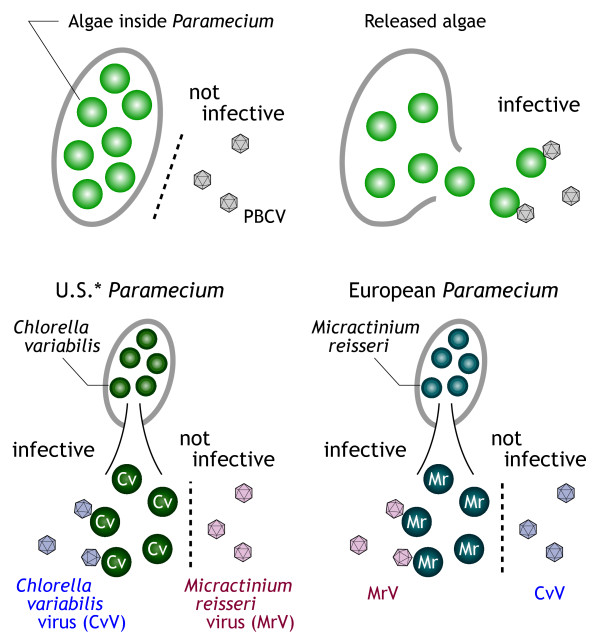
**Schema of PBCV infection of the symbiotic algae of *Paramecium***. *Paramecium possessing *Chlorella variabilis *has been reported in Japan, China, and Australia as well as the United States.

*Chlorella *virus that infects *Chlorella*-like algae symbiotic with coelenterate *Hydra viridis *was first discovered in 1981 and designated HVCV (*Hydra viridis Chlorella *virus) [1]. Subsequently, another *Chlorella *virus that infects *Chlorella*-like algae symbiotic with ciliate *Paramecium bursaria *was described (*Paramecium bursaria Chlorella *virus [PBCV]) [2]. Studies on HVCV and PBCV have revealed strong host-parasite relationships [[3] and references therein]: HVCVs do not infect *P. bursaria *symbionts, whereas PBCVs do not infect hydra symbionts; PBCVs collected in the United States infect algal strain NC64A (representative of U.S. *P. bursaria *symbionts) and other U.S. *P. bursaria *symbionts, but they do not infect algal strain Pbi (representative of German *P. bursaria *symbionts) or other European *P. bursaria *symbionts; PBCVs collected in Europe infect European *P. bursaria *symbionts but do not infect U.S. *P. bursaria *symbionts (Fig. 1). Later, another group of viruses that infect *Chlorella*-like algae symbiotic with heliozoon, *Acanthocystis turfacea *was described [4]. *Chlorella *viruses studied to date, therefore, can be divided into four categories: HVCV, NC64A virus, Pbi virus, and ATCV (*Acanthocystis turfacea Chlorella *virus). Furthermore, none of the *Chlorella *viruses infect free-living green algae, and NC64A viruses exhibit a degree of diversification with regard to, for example, plaque size, hyaluronan productivity, and DNA methylation level. Note that viruses attack isolated (or released) algae but not algae inhabiting their hosts (i.e., hydra or paramecium).

Recent taxonomic studies on *P. bursaria *symbionts indicated that the algal group "American" containing strain NC64A and the algal group "European" containing strain Pbi are genetically distinct from each other, as well as from any known free-living algae and other symbiotic algal species [5]. Consequently, each group has been given a distinct species name, *Chlorella variabilis *("American") and *Micractinium reisseri *("European") [6]. Due to the defects in taxonomy of the host algae, circular virus names (i.e., *Hydra viridis Chlorella *virus [HVCV], *Paramecium bursaria Chlorella * [PBCV], and *Acanthocystis turfacea Chlorella *virus [ATCV]) and strange names based on host strains (i.e., NC64A virus and Pbi virus) have been used. In this report, viruses infecting *C. variabilis *and *M. reisseri *are referred to as *C. variabilis *virus (CvV) and *M. reisseri *virus (MrV), respectively (Fig. 1).

*Chlorella variabilis *F36-ZK isolated from Japanese *P. bursaria *[7] and *M. reisseri *SW1-ZK isolated from German *P. bursaria *[8] are lesser-known hosts in PBCV studies, although they are well researched strains in phylogenetic studies [9,10]. We carried out a screen for viruses from Lake Biwa and adjacent water environments using *C. variabilis *F36-ZK and *M. reisseri *SW1-ZK as hosts. Here, we present the results of the environmental study and the results of a biological study of one strain, CvV-BW1, obtained in the environmental study.

## Methods

### Algal strains and culture conditions

*Chlorella variabilis *F36-ZK (NIES-2540) and NC64A (ATCC 50258) were cultured in C liquid medium [11] with 200 mg L^-1 ^arginine, while *M. reisseri *SW1-ZK was cultured in C liquid medium with 1 g L^-1 ^casamino acid. They were maintained under fluorescent illumination (16 L:8 D, 50 μmol photons m^-2 ^s^-1^) at 25°C.

### Detection of viruses

Water samples were collected from eight sites at Lake Biwa (the largest lake in Japan) and the adjacent Lake Yogo. For four sites at Lake Biwa, sampling was carried out almost every month to observe seasonal variations in the virus populations. Water samples were centrifuged at 48,000 × *g *for 30 min, and then virus concentrated waters were filtrated through nitrocellulose membrane (pore size, 0.45 μm). Whether cultures contained the viruses was determined by mixing with *C. variabilis *F36-ZK or *M. reisseri *SW1-ZK liquid cultures on 48-well microplates. The titers (PFU mL^-1^) of virus-containing cultures were determined by serial dilution.

### Plaque assay and virus isolation

We followed a previously described plaque assay procedure [12] using C medium with 5 g L^-1 ^glucose and 200 mg L^-1 ^serine (CGS) in place of modified Bold's basal medium (MBBM). Plaques were observed after 3 days of cultivation. Single plaques were picked up and transferred to fresh algal lawn plates. Single virus strains were established by repeating this procedure several times.

### Electric microscopic observation

*Chlorella variabilis *was incubated for 2 h (25°C) after adding cultured virus, then fixed with 3% glutaraldehyde and subsequently with 0.5% osmic acid. Resin-embedded specimens were cut into ultrathin sections, stained with 3% uranyl acetate, and then observed under an electron microscope at an acceleration voltage of 75 kV.

Another culture was centrifuged at 5000 × *g *for 5 min, and the resulting supernatant was dropped onto Veco H-200 mesh (Electron Microscopy Sciences, Hatfield, PA, USA), stained with 1% uranyl acetate, and then observed at 75 kV.

### SDS-PAGE analysis

*Chlorella variabilis*-CvV-BW1 culture mixture was first centrifuged at 12,000 × *g *for 10 min to remove algal debris, and the supernatant was centrifuged at 37,000 × *g *for 1 h to precipitate virus particles. Urea was added to the precipitate at a final concentration of 4 M. After incubation at 45°C for 1.5 h, the mixture was centrifuged at 37,000 × *g *for 10 min to remove the precipitate. The supernatant was subjected to standard SDS-PAGE analysis; 4.5% and 7.5% polyacrylamide gels were used for condensation and separation, respectively. Electrophoresis was performed at a constant voltage of 200 V using a tank buffer consisting of 0.1% SDS, 192 mM glycine, and 25 mM Tris.

### N-terminal amino acid sequence analysis and amino acid sequence homology search

After SDS-PAGE, proteins in the polyacrylamide gels were electroblotted onto polyvinylidene fluoride membranes (Amersham Biosciences, Piscataway, NJ, USA) using a Horizeblot apparatus (Atto, Tokyo, Japan) at a constant current of 0.8 mA cm^-2 ^for 1 h. After staining the membrane with 0.1% Ponceau solution, bands of interest were cut out and subjected to N-terminal amino acid sequencing using a PPSQ-21/23 peptide sequencer (Shimadzu, Kyoto, Japan); in the present study, 15 N-terminal amino acids were examined. Using the obtained 15 amino acid sequence, a homology search was carried out using NCBI protein-protein BLAST http://www.ncbi.nlm.nih.gov/BLAST/.

### Pulsed-field gel electrophoresis (PFGE)

An equal volume of 1.4% InCert Agarose (45°C; Bio-Rad, Hercules, CA, USA) was added to a suspension of *Chlorella *virus, and the mixture was poured into a mold and solidified by cooling at room temperature. An agar block was removed from the mold, soaked in cell wall-dissolving solution (1 mg mL^-1 ^proteinase K, 1% lauroyl sarcosinate, 0.01 M Tris-HCl, pH 8.0), and incubated at 50°C for 16 h. The mixture was discarded, and fresh mixture was supplied and incubated at 50°C for 24 h. After incubation at 4°C for 2 days in TE buffer (10 mM Tris-HCl, pH 8.0, containing 0.1 mM EDTA), the gel block was subjected to PFGE using 1% Seakem GTG agarose (Bio-Rad) and a CHEF-DRIII system (Bio-Rad). Tank buffer (89 mM Tris-HCl, pH 8.0, containing 2 mM EDTA and 89 mM boric acid) was used. Electrophoresis was performed at 14°C. Other conditions were as follows: switching time, 22 to 50 s; total time, 24 h; voltage, 6.6 V cm^-1^. *Saccharomyces cerevisiae *chromosomes (Bio-Rad) and λ DNA ladder (Bio-Rad) were used as size markers.

### Extraction of CvV-BW1 DNA

Five units of DNase I was added to the virus particles (precipitate) described above. The resultant precipitate was suspended, and the suspension was incubated at 37°C for 1 h. Proteinase K to at a final concentration of 1 mg mL^-1^, EDTA to 0.1 M, and SDS to 0.5% were then added to the suspension. After incubation at 60°C for 1 h, the mixture was subjected to the standard phenol extraction procedure [13].

### Digestion of CvV-BW1 DNA with restriction enzymes

Restriction enzymes were purchased from Takara Bio (Otsu, Japan) and/or Nippon Gene (Tokyo, Japan). Restriction enzymes were used under the conditions recommended by the manufacturers.

### HPLC analysis of methylated nucleotides

CvV-BW1 DNA was mixed with Nuclease P1 (GC Analysis Standard Kit; Yamasa, Choshi, Japan). The mixture was incubated at 50°C for 1 h. After digestion, the mixture was subjected to HPLC using a column of ODS-YMC PACK AQ-312 (6.0 mm in inner diameter and 150 mm in length) (YMC, Kyoto, Japan). HPLC conditions and peak assignment were adopted from Kowalak et al. [14] and Ushida et al. [15].

### Hyaluronan labeling

Hyaluronan labeling was performed according to a modification of the technique reported by Graves et al. [16] and Cohen et al. [17]. *Chlorella variabilis *F36-ZK was incubated for 2 h (25°C) after adding viruses, of which 200 μL was centrifuged at 5000 × *g *for 5 min. Cells were fixed in phosphate-buffered saline (PBS) with 3% paraformaldehyde for 20 min. Centrifugation and PBS wash were repeated three times. Then, cells were incubated for 2 h at 37°C with 20 μL of biotinylated hyaluronic acid binding protein (bHABP, 0.5 mg mL^-1^; Seikagaku, Tokyo, Japan). Centrifugation and PBS wash were repeated three times, followed by incubation with 50 mL of CY3-conjugated streptavidin (1.8 mg mL^-1^; ENCO, Petach Tikva, Israel) for 30 min at 37°C. Centrifugation and PBS wash were repeated three times, and then cells were observed under a fluorescence microscope with excitation at 510 to 550 nm.

### DNA polymerase gene analyses

The DNA polymerase gene (*dnapol*) region was amplified using the forward primer M37dpo0310F (5'-CAA TGG TGC AAT TCG TGT TC-3') and reverse primer M37dpo2390R (5'-GTG AAT TTT TCC ATG GGA TAC TC-3'). These primers were designed with reference to three longer determined sequences of PBCV-1 (M86836), NY-2A (M86837), and CVK2 (AB011500). A standard three-step PCR protocol was carried out (annealing temperature of 55°C) using Takara Ex Taq (Takara Bio) according to the manufacturer's directions. The PCR product was confirmed by agarose gel electrophoresis, purified by polyethylene glycol (PEG) precipitation, and then sequenced directly.

The obtained sequence was compared to those of *Chlorella *viruses available in the databases. The alignment was performed with reference to Zhang et al. [18], and 663 nucleotide positions (Polymerase Domain, excluding introns) contributed to phylogenetic analysis. Phylogenetic tree was constructed by the neighbor-joining (NJ) methods of Saito and Nei's evolutionary model using Clustal X ver. 2 [19]. The significance of each node was tested using 1000 bootstrap replicates. Evolutionary divergence between sequences was estimated using the Jukes-Cantor method in MEGA4 [20].

## Results and discussion

### Ecological studies of viruses in Lake Biwa

**Figure 2:**
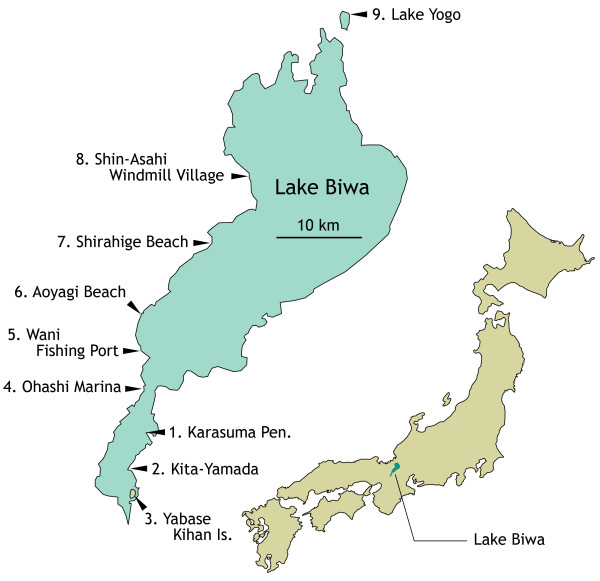
**Locations of sampling sites**. Sites numbered 1 to 4 were surveyed for seasonal transition.

Using two strains of algae, *C. variabilis *F36-ZK and *M. reisseri *SW1-ZK, we surveyed algae-lytic viruses at nine sites in Lake Biwa and Lake Yogo, both in Shiga Prefecture, western Honshu, Japan (Fig. 2), between May and July 2004. At all sites and at nearly all sampling time points, we detected viruses infecting *C. variabilis*. None of the isolated viruses infected *M. reisseri *in this study, indicating that all of those obtained were *C. variabilis *virus (CvV = NC64A virus).

Since the development of a screening method for virus sampling [12], both CvV and MrV have been detected from extensive regions of the world, but MrV has never been recorded from East Asia [21,22]. In the present study, we also found CvVs, but not MrV, from the water of Lake Biwa. Van Etten [21] indicated that the factors influencing the distribution patterns of these viruses are probably latitude and altitude. Based on a series of taxonomic studies on symbiotic algae, the all *P. bursaria *collected so far in Japan have been verified as *C. variabilis*-harboring type [6]. The absence of MrV in Lake Biwa is inevitable if no *M. reisseri *occur in this lake.

The results of our ecological studies are summarized in Table 1. The titers of CvVs were mostly between 0.5 and 50 PFU mL^-1^. This density level is the same or slightly lower than those reported in previous studies [e.g., [23,24]]. Exceptionally high values were recorded in May (85.3 PFU mL^-1^) and June (171.0 PFU mL^-1^) 2004 at Shin-Asahi Windmill Village (site 8). In addition, no clear seasonal changes in population density were detected, and the population densities were particularly low (< 1.5 PFU mL^-1^) in high-temperature waters (around 30°C) in July 2004 at all the sites except Shin-Asahi Windmill Village.

**Table 1:** Seasonal transition of *Chlorella variabilis *viruses concentration (PFU mL^-1^) for nine sampling sites

Sampling date	Water temp. (°C)	Site 1	Site 2	Site 3	Site 4	Site 5	Site 6	Site 7	Site 8	Site 9
2004 May	--	2.67	21.3	21.3	2.67	21.3	2.67	ND	85.3	21.3
June	16.5-19.5	21.3	5.33	10.7	5.33	5.33	--	10.7	171.0	42.7
July	29.3-32.0	ND	0.67	0.67	ND	1.33	--	ND	10.7	0.67
Sept.	24.0-25.5	0.67	5.33	10.7	10.7	--	--	--	--	--
Oct.	16.0-16.9	0.67	1.33	10.7	5.33	--	--	--	--	--
Nov.	10.2-12.0	5.33	5.33	5.33	5.33	--	--	--	--	--
Dec.	8.8-11.2	0.67	1.33	21.3	0.33	--	--	--	--	--
2005 Jan.	3.9-6.8	21.3	1.33	0.67	0.67	--	--	--	--	--
Feb.	5.2-8.3	1.33	1.33	10.7	5.33	--	--	--	--	--
Mar.	8.0-9.4	5.33	10.7	21.3	5.33	--	--	--	--	--
Apr.	17.0-18.8	5.33	21.3	21.3	5.33	--	--	--	--	--
June	23.0-24.0	5.33	10.7	42.7	10.7	--	--	--	--	--

Sampling Sites: 1. Karasuma Pen., 2. Kita-Yamada, 3. Yabase Kihan Is., 4. Ohashi Marina, 5. Wani Fishing Port, 6. Aoyagi Beach, 7. Shirahige Beach, 8. Shin-Asahi Windmill Village, 9. Lake Yogo (also see Fig. 2). ND: Not detected. --: Not determined.

Reisser et al. [25] attempted to explain the density of viruses in natural water environments; the viral density depends on the *P. bursaria *population and the probability of its burst (i.e., release of symbiotic algae). In 2003 and 2004, a major outbreak of koi herpes virus (KHV) occurred in Japan. Populations of koi (common carp) in Lake Biwa were attacked by the virus from May to June 2004, which caused mass death of the fish. Large numbers of koi carcasses washed ashore onto the coastal area of a sampling point, Shin-Asahi Windmill Village (site 8). At this time, shallow water around this point seemed to be under low-oxygen conditions caused by decomposition of fish carcasses. We detected the highest virus concentrations at this sampling point at these times. In contrast, lower densities of viruses were common in July 2004 at all sampling points (Table 1). In general, high temperature and strong light prompt *Paramecium *to avoid its translatory movement. Low oxygen levels may have caused bursting of some *P. bursaria *cells, with summer heat prompting the migration of *P. bursaria*.

### Plaque-forming assay

**Figure 3:**
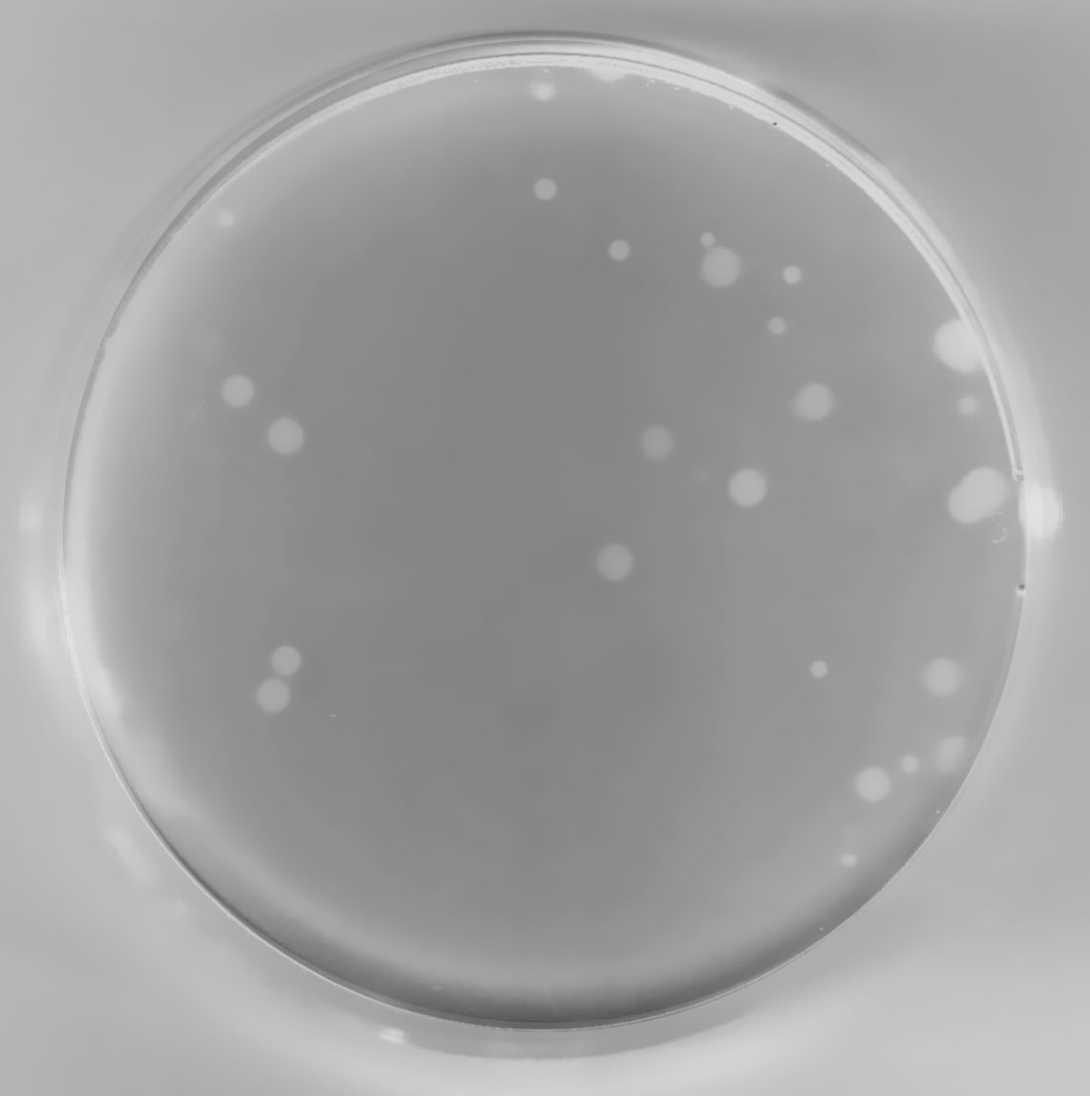
**Plaque formation on the *Chlorella variabilis *lawn plate (90 mm petri dish)**. Both large and small plaques were seen.

**Figure 4:**
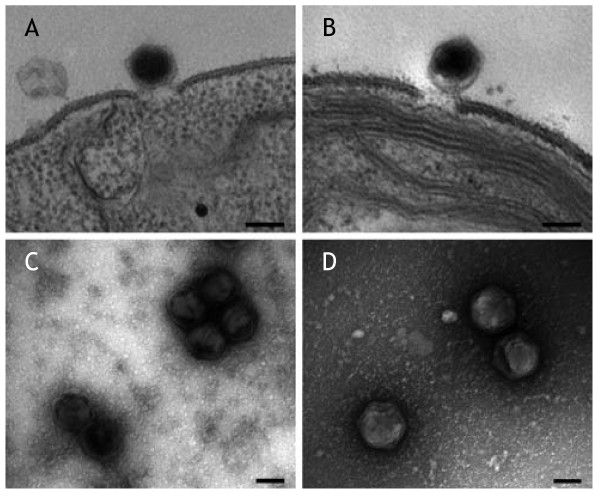
**Polyhedral particles, attaching to the external surface of the algal cell wall (TEM, upper panel) and released particles (SEM, lower panel) of *Chlorella variabilis *virus**. CvV-BW1 is on the left (A and C) and CvV-BW3 is on the right (B and D). Scale bars are 100 nm.

We performed plaque-forming assay of the viruses, and all but one plate revealed plaques 3 to 4 mm in diameter. The exceptional plate, for the sample water from Ohashi Marina (site 4, May 2004), had smaller plaques (about 1 mm in diameter) in addition to the normal-sized plaques (Fig. 3). Viruses recovered from one of the smaller plaques formed smaller plaques on reinfection. By repeating this procedure several times, we concluded that we had established a pure clone of smaller plaque-forming virus, which we designated CvV-BW1. We subsequently focused our attention on the biological characteristics of CvV-BW1. We used four independent clones of normal-sized plaque-forming viruses, CvV-BW2, -BW3, -BW4, and -BW5, obtained in the same ecological study. These CvV-BW strains infect *C. variabilis *NC64A but not *M. reisseri*. Similar to known CvVs, CvV-BW1 appeared as polyhedral particles about 150 nm in diameter (Fig. 4).

### Protein of CvV-BW1

**Figure 5:**
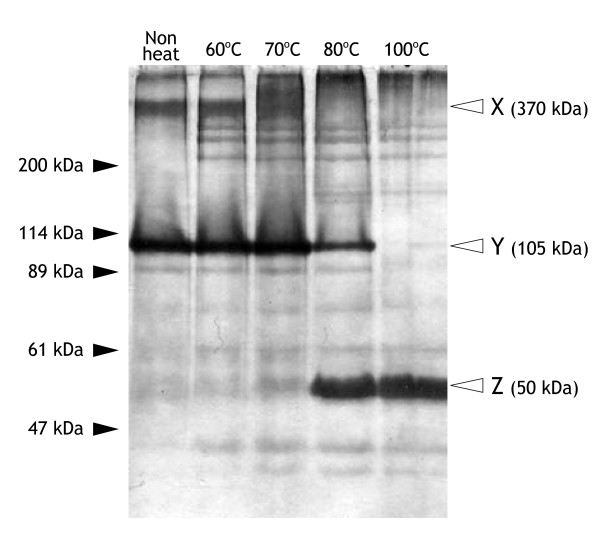
**SDS-PAGE analysis of CvV-BW1 virion proteins**. From the left, no heat treatment, heat treatment at 60°C, at 70°C, at 80°C, at 100°C prior to electrophoresis.

First, we analyzed the protein composition of CvV-BW1 by SDS-PAGE. As shown in Fig. 5, when viral proteins were not heat-treated (leftmost lane), two major bands, designated X and Y, were observed. Judging from the intensity, the proteins in these bands accounted for 80% of the total viral proteins. By increasing the temperature of the heat treatment to 70°C, band X faded, whereas the intensity of band Y increased. With further increases in temperature, band Y faded, whereas the intensity of band Z increased; with heat treatment at 100°C, only band Z was observed. The sizes of proteins in bands X, Y, and Z were estimated to be 370 kDa, 105 kDa, and 50 kDa, respectively, compared to size markers. To identify proteins of these bands, we performed an N-terminal amino acid sequence analysis. Although we did not obtain meaningful results for protein of band X, presumably due to an insufficient amount of protein, we obtained the same sequence, AGGLSQLVAYGAQDV, for the proteins recovered from bands Y and Z. The obtained N-terminal amino acid sequence was completely identical to those of the major capsid proteins of all PVCVs (NA46A virus and Pbi virus) reported to date. Therefore, we concluded that CvV-BW1 has a major capsid protein of 50 kDa. We thus contended that band Y represent dimmer of the 50 kDa major capsid protein. The assignment of protein of band Z remained to be established. In addition, CvV-BW1 showed at least nine distinct bands, which showed no changes in electrophoretic mobility according to heat treatment conditions. Further studies are required to characterize the proteins corresponding to these bands.

### Size of CvV-BW1 DNA

**Figure 6:**
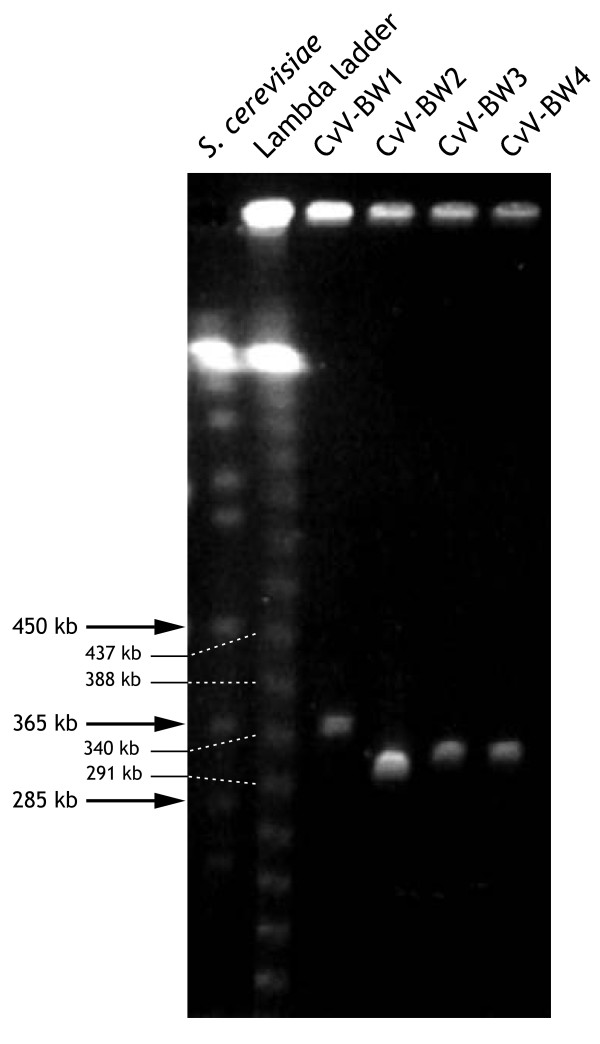
**Estimates of virion genome sizes**. From the left, *Saccharomyces cerevisiae *chromosomes (Bio-Rad), λ DNA ladder (Bio-Rad), CvV-BW1, CvV-BW2, CvV-BW3, and CvV-BW4.

To estimate the size of CvV-BW1 DNA, we carried out pulsed-field gel electrophoresis as described in the Methods section. The results are shown in Fig. 6. Compared to *Saccharomyces cerevisiae *chromosomes and λ DNA ladder, we concluded that the CvV-BW1 DNA is 370 kb in length, assuming that it has a linear DNA genome. CvV-BW1 DNA was somewhat larger than those of CvV-BW2, -BW3, and -BW4.

### Resistance/susceptibility of CvV-BW1 DNA to restriction enzymes

**Figure 7:**
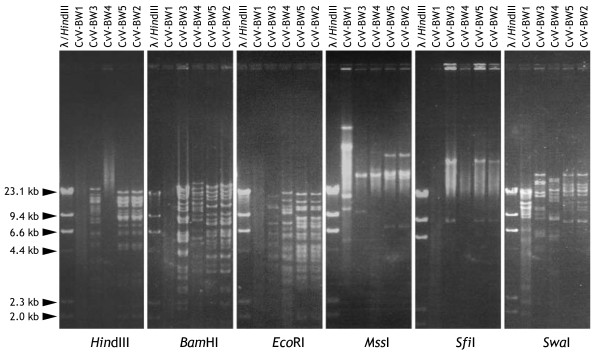
**Restriction enzyme digestion of CvV-BW virion genomes**.

CvV has been divided into 16 "species" based on the restriction enzyme digestion patterns and various other characteristics [3]. We attempted to cut the DNA of CvV-BW strains using six widely used restriction enzymes: *Hin*dIII, *Bam*HI, *Eco*RI, *Mss*I, *Sfi*I, and *Swa*I (Fig. 7). The results indicated that CvV-BW1 DNA was much more resistant to cleavage than the DNAs of other BW strains. That is, CvV-BW1 DNA was cut only by *Mss*I and *Swa*I, while CvV-BW2, -BW3, and -BW5 DNAs were effectively cut by all six enzymes tested. DNAs of CvV-BW2 and -BW5 showed the same band pattern, indicating that they are clones of a single species.

**Figure 8:**
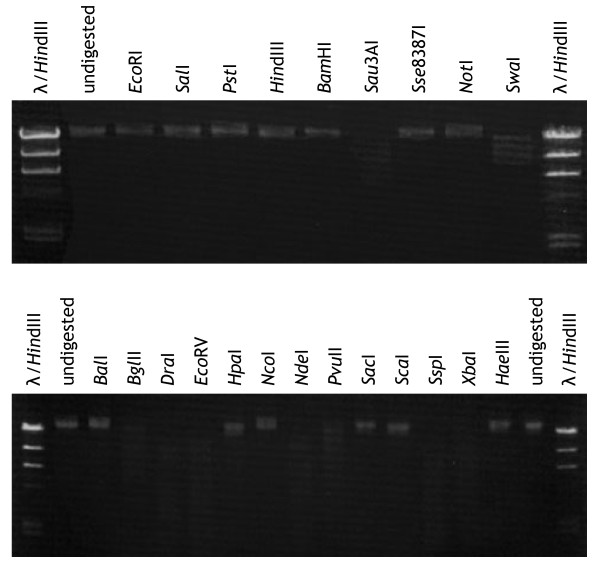
**Restriction enzyme digestion of the CvV-BW1 genome**. For band sizes of the λ/*Hin*dIII marker, see Fig. 7. A summary of the effectiveness is shown in Tables 2 and 3.

An additional 18 restriction enzymes were tested for CvV-BW1 DNA; 11 of the enzymes did not effectively cut CvV-BW1 DNA (Fig. 8). The enzymes that did not effectively cut CvV-BW1 DNA are listed in Table 2 (Enzymes I), while those that cut CvV-BW1 DNA are shown in Table 3 (Enzymes II). Van Etten et al. [3] classified CvV DNAs into 11 restriction groups (A to K) based on the effects of 13 restriction enzymes. Although the enzymes they used were not identical to those applied here, some were common to the two studies. Judging from the cleavage patterns with the common enzymes, we concluded that CvV-BW1 DNA belongs to group H, which is characterized by resistance to *Eco*RI but susceptibility to *Bgl*II.

**Table 2:** Restriction enzymes that did not effectively cut CvV-BW1 DNA (Enzymes I)

Restriction enzyme	Recognition sequence
*Bal*I	TGGCCA
*Bam*HI	GGATCC
*Eco*RI	GAATTC
*Hae*III	RGCGCY
*Hin*dIII	AAGCTT
*Hpa*I	GTTAAC
*Nco*I	CCATGG
*Not*I	GCGGCCGC
*Pst*I	CTGCAG
*Pvu*II	CAGCTG
*Sac*I	GAGCTC
*Sal*I	GTCGAC
*Sca*I	AGTACT
*Sfi*I	GGCCNNNNNGGCC
*Sse*8387I	CCTGCAGG

**Table 3:** Restriction enzyme that effectively cut CvV-BW1 DNA (Enzymes II)

Restriction enzyme	Recognition sequence
*Bgl*II	AGATCT
*Dra*I	TTTAAA
*Eco*RV	GATATC
*Nde*I	CATATG
*Mss*I	GTTTAAAC
*Sau*3AI	GATC
*Ssp*I	ACTAGT
*Swa*I	ATTTAAAT
*Xba*I	TCTAGA

Analysis of the enzymes of I and II indicated that the AT/GC ratio of the recognition sequences was quite different between them; enzymes I were rich (almost 65%) in GC, whereas enzymes II were rich (75%) in AT. This result can be rationalized in two ways: CvV-BW1 DNA is rich in AT and poor in GC or CvV-BW1 DNA is highly modified at G and/or C. Nucleotide sequence analysis of clones in the CvV-BW1 genome library did not reveal any evidence that CvV-BW1 DNA was AT-rich; according to our preliminary genome analysis, the GC content of CvV-BW1 is in the vicinity of 41.3%. Therefore, we suspected that CvV-BW1 DNA would have a high incidence of G and/or C modification. To confirm this speculation, we examined the frequencies of modified nucleotides in CvV-BW1 DNA; the results revealed 33.2% 5 mC relative to 5 mC+C and 31.0% 6 mA relative to 6 mA+A.

### Production of hyaluronan by CvV-BW1

**Figure 9:**
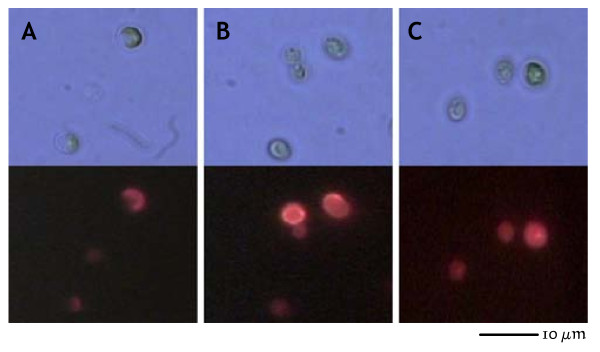
**Light (upper) and fluorescence (lower) images of *Chlorella variabilis***. A: Noninfected algae. Slight fluorescence assumed to be intrinsic fluorescence of the chloroplast; B: CvV-BW1-infected algae; C: CvV-BW3-infected algae.

The best characterized CvV, PBCV-1, encodes hyaluronan synthase (HAS), which functions in the production of hyaluronan, a polysaccharide covering the outside of the algal cell wall [26]. Graves et al. [16] showed that some CvVs produce hyaluronan during infection, although others do not [27]. Therefore, we examined whether CvV-BW1 produces hyaluronan. Algal cells showed strong fluorescence 120 min after infection of CvV-BW1 (stronger than those infected by CvV-BW3) using the streptavidin-biotin system, indicating the production of hyaluronan by CvV-BW1 (Fig. 9).

### DNA polymerase gene phylogeny of CvV-BW1

**Figure 10:**
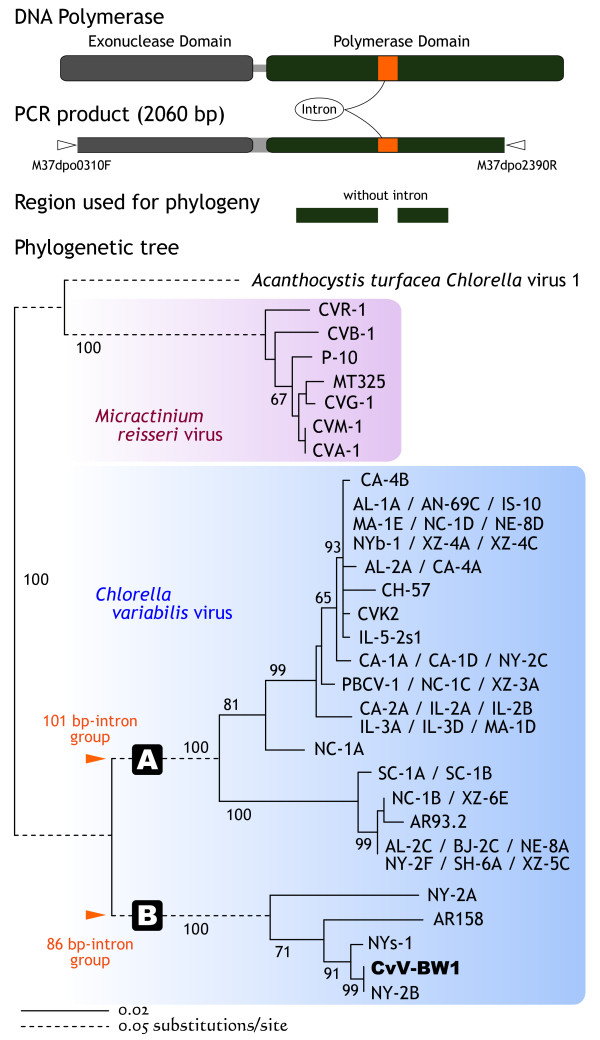
**Domain structure of the *dnapol *gene, obtained sequence, and neighbor-joining tree of PBCVs based on *dnapol *gene sequences**. *Chlorella variabilis *virus split into two lineages, A (101 bp intron group) and B (86 bp intron group). Numbers at major nodes represent bootstrap probabilities (1000 replicates).

The DNA polymerase genes, *dnapol*, of viruses appear to have evolved from a common ancestral gene, and are highly conserved within the viral family Phycodnaviridae [28,29]. Therefore, we attempted to amplify a homolog from CvV-BW1 via PCR using sequences that are common to nearly all strains, with PBCV-1, NY-2A, and CVK2 as primers (Fig. 10). The amplification fragment of 2060 bp obtained by PCR was then sequenced (AB572585). Multiple alignment with the known PBCV *dnapol *sequences indicated that this 2060-bp fragment contained an intron of 86 bp. Introns of the same length are present in *dnapol *of AR-158, NY-2A, NY-2B, and NYs-1 [18]. In the phylogenetic tree constructed from the exon regions of *dnapol*, CvV was found to be divided into two clades, A and B, with a minimum distance of 0.237 between these clades. As shown in Fig. 10, all CvVs with the 86-bp intron belonged to the same group that included CvV-BW1 affiliated to clade B, while all CvVs affiliated to clade A possessed an intron of 101 bp in their *dnapol *genes.

### Identity of CvV-BW1

Van Etten et al. [3] reported that three viral strains, CA-4A, XZ-4A, and XZ-5C, belong to restriction group H. Note that these strains all form small plaques (1 mm in diameter) and are rich in methylated nucleotides (40% to 45% 5 mC among C+5 mC, and 20% to 30% 6 mA among A+6 mA). As presented above, CvV-BW1 shares these properties. However, all the strains belonged to *dnapol *clade A (101-bp intron group) (Fig. 10). Members of *dnapol *clade B (86-bp intron group) differ from CvV-BW1 in some respects. NY-2A belongs to restriction group I, NYs-1 belongs to group F, and NY-2B belongs to group G. Although the restriction group of AR158 has not been determined, AR158 does not encode HAS [30]. Taken together, these findings indicate that CvV-BW1 does not belong to any of the 16 CvV "species" defined to date.

## Conclusions

We detected *C. variabilis *virus (NC64A virus) but not *M. reisseri *virus (Pbi virus) in the water of Lake Biwa, Japan. The highest virus density was recorded in water under low-oxygen conditions, whereas lower virus densities were commonly found in the seasons when the lake waters reached up to around 30°C. These results suggest that viral density is affected by the population density of *P. bursaria *and its burst ratio.

The viral strain CvV-BW1 found in Lake Biwa was examined in detail with regard to plaque size, electron microscopic features, protein composition, genome size, restriction enzyme digestion, level of DNA methylation, production of hyaluronan, and phylogeny of the DNA polymerase gene. Taken together, all of these observations indicate that CvV-BW1 is likely to be a new species of *C. variabilis *virus.

## List of abbreviations used

CvV: *Chlorella variabilis *virus; MrV: *Micractinium reisseri *virus; PBCV: *Paramecium bursaria Chlorella *virus.

## Competing interests

The authors declare that they have no competing interests.

## Authors' contributions

MS screened and isolated the viral strains, and then tested hyaluronan productivity. YK observed viruses by electron microscopy. SiU carried out the protein analysis. YM examined the viral genome sizes, and then MS and YM confirmed the results of restriction enzyme digestion. YH examined viral DNA modification. RH contributed to DNA polymerase gene analyses. RH and BiO prepared the manuscript. NI initially conceived of this study and RH, MK, BiO, NI finalized the experimental design. All authors have read and approved the final manuscript.

## Authors' information

1 Department of Biomedical Science, College of Life Sciences, Ritsumeikan University, Noji Higashi 1-1-1, Kusatsu, 525-8577 Japan.

2 Department of Bioscience and Biotechnology, Faculty of Science and Engineering, Ritsumeikan University, Noji Higashi 1-1-1, Kusatsu, 525-8577 Japan.

3 Department of Biotechnology, College of Life Sciences, Ritsumeikan University, Noji Higashi 1-1-1, Kusatsu, 525-8577 Japan.

4 Department of Pharmacy, College of Pharmaceutical Sciences, Ritsumeikan University, Noji Higashi 1-1-1, Kusatsu, 525-8577 Japan.
